# Industrial pilot‐scale study on the ripening and quality parameters of Toma Piemontese PDO raw milk cheese in ozone‐controlled atmosphere

**DOI:** 10.1002/jsfa.70013

**Published:** 2025-06-25

**Authors:** Vanessa Eramo, Cristian Carboni, Roberto Forniti, Rinaldo Botondi

**Affiliations:** ^1^ Department for Innovation in Biological, Agro‐Food and Forest Systems University of Tuscia Viterbo Italy; ^2^ De Nora Water Technologies Italy Milan Italy

**Keywords:** non‐thermal technology, dairy production, gaseous ozone, raw milk cheese, spoilage microflora

## Abstract

**BACKGROUND:**

Non‐thermal technologies are becoming of high interest in dairy production as sustainable tools to enhance food safety. Raw milk cheeses present microbiological challenges due to spoilage and the absence of real thermal treatments. This study investigates an industrial application of gaseous ozone to control spoilage microflora on Toma Piemontese PDO raw milk cheese rind during ripening to preserve the overall quality.

**RESULTS:**

The test was conducted on ‘Toma’ cheese over 60 days. An automated ozone prototype was installed in a cheese‐storing ripening room, and two pilot batch tests were designed: (i) untreated cheese wheels in air ripening room (CTL) and (ii) cheese wheels exposed to low‐concentration gaseous ozone atmosphere (O_3_, 400 ppb, 0.856 mg m^−3^ in air) for 6 h per night, three times a week, until day 40 of ripening. Thanks to an attempted operational design, treatments were scheduled at night to ensure operator safety. Ozone significantly reduced microbial growth from the first 10 days of ripening, improving microbiological and visual quality. Cheese sensory quality was assessed: the O_3_ sample showed acceptance scores similar to or higher than the CTL sample, particularly for the rind appearance and color. Both samples exhibited a high perception of milky notes, in line with Protected Designation of Origin (PDO) specifications. No issues related to undesirable compounds linked to lipid peroxidation were observed, as confirmed by stable peroxide values over time.

**CONCLUSION:**

This industrial scalability test suggests that ozone technology can enhance food safety in dairy production and possibly offer advantages in terms of environmental and economic sustainability. © 2025 The Author(s). *Journal of the Science of Food and Agriculture* published by John Wiley & Sons Ltd on behalf of Society of Chemical Industry.

## INTRODUCTION

Recent advancements in food technology drive innovation in the dairy field, particularly in developing sustainable methods for enhancing product safety and quality. Food technology has long played a crucial role in improving human wellbeing, and growing awareness of the environmental and economic impacts of conventional preservation methods, such as heat sterilization and use of chemical preservatives, has further highlighted the need for more sustainable solutions.^1^ The traditional approaches, while effective, can compromise the sensory and nutritional properties of foods, prompting a shift towards technologies with less impact on quality. In this context, non‐thermal technologies have gained attention for their ability to control microbial growth while preserving products' overall quality, reducing energy consumption, and improving shelf‐life extension.^2^

As the demand for more efficient and eco‐friendly food production processes grows, gaseous ozone treatment represents a promising solution for addressing common challenges in cheese ripening, especially in controlling spoilage microflora on the rind.^3^, ^4^, ^5^ Ozone, with oxidation potential of −2.07 V, is recognized as safe by the Food and Drug Administration (FDA).^6^ In Europe ozone generated from oxygen was approved on 2 June 2023 as an active substance for use in biocidal products of product‐types 2, 4, 5 and 11 in accordance with Regulation (EU) No 528/2012 of the European Parliament and of the Council.^7^ Product‐type 4 concerns biocides used for the disinfection of equipment, containers, consumption utensils, surfaces, or pipework associated with the production, transport, storage, or consumption of food or feed (including drinking water) for humans and animals or used to impregnate materials that might enter into contact with food. Ozone is generated on‐site and it has a short half‐life with no release of harmful residues in the environment while ensuring its efficiency in preserving food products. Ozone's oxidative properties require strict safety limits with high‐concentration treatments recommended in worker‐free periods.^8^

In particular, the application of gaseous ozone in dairy production is aligned with the growing trend toward sustainability in the agri‐food sector. The concept of ‘One Health’, which emphasizes the interconnectedness of human, animal, and environmental health, supports the adoption of innovative technologies to minimize the environmental footprint of food production while maintaining its safety and quality.^9^ Furthermore, ozone, unlike other chemical products, and in particular antibiotics, does not create microbial resistance phenomena. Protected Designation of Origin (PDO) certifications are an important pillar in sustainable agri‐food systems, providing economic and ecological benefits. In 2024, Italy remains a global leader in the PDO sector, with 584 registered products, underscoring their value in preserving agrobiodiversity and promoting the sustainable development of rural economies.^10^ The ecological relevance of PDO products is evident in their contribution to reducing the carbon footprint through localized production, protecting autochthonous microbiota biodiversity, and maintaining traditional production practices tied to specific territories.

‘Toma Piemontese’ is one of the traditional Italian raw cow's milk cheeses under a PDO certification^11^ whose ripening process is essential for the development of its distinctive sensory profile due to biochemical changes that can occur in this phase.^12^ The uncontrolled mold growth can lead to excessive proteolysis and lipolysis, negatively affecting the cheese rind and its overall quality. As a result, mold proliferation in cheese can result in significant industrial and commercial financial losses^13^ and in some cases it can also lead to the presence of mycotoxins on the crust.^8^ Generally, the use of raw milk in different dairy production introduces specific challenges during the aging process, particularly in controlling the growth of spoilage microflora. Traditional methods like manual turning and brushing, while effective to some extent, are labor‐intensive and limited in their ability to manage microbial proliferation.^4^

Although cheese made with raw milk can be produced safely, there have been foodborne illness outbreaks associated with various types of raw milk cheeses, caused by *Salmonella*, *Campylobacter*, *Staphylococcus aureus*, *Escherichia coli* O157:H7, and *Listeria monocytogenes*.^14^, ^15^ These challenges highlight the need for innovative solutions that ensure safety without compromising the unique qualities of dairy products. In this context, non‐thermal ozone technology offers a promising alternative. The application of gaseous ozone treatment has been explored, demonstrating potential in addressing the challenges associated with raw milk cheeses and a cleaner environment.^16^, ^17^ Laboratory‐scale studies have already shown the effectiveness of gaseous ozone at low concentrations in controlling spoilage microflora on Toma Piemontese cheese rind while preserving the sensory and chemical cheese properties.^5^, ^18^

Building on these laboratory‐scale findings, the current research focuses on scaling up this technology to an industrial pilot‐scale. The transition aims to validate the effectiveness of gaseous ozone treatment at low concentrations in real‐world production settings, ensuring its scalability and practical benefits for the ripening of Toma Piemontese cheese. By integrating an automated ozone prototype into traditional cheese‐making practices, this innovative approach seeks to ensure the quality and safety of raw milk cheeses, extend shelf life, reduce waste, and align with sustainability principles, offering a viable solution for modern dairy production while maintaining traditional processes.

## MATERIALS AND METHODS

### Experimental design and gaseous ozone treatments

Following laboratory tests to evaluate the effectiveness of gaseous ozone in controlling microorganisms affecting Toma Piemontese PDO cheese preserving the quality characteristics, an industrial pilot‐scale test was carried out to assess the feasibility and scalability of using gaseous ozone technology in dairy production environments (S‐O_3_‐SDairy, I TESORI DELLA TERRA s.c.a.s. ONLUS, Cervasca, Italy). A prototype device (design, assembly, and fine‐tuning) was created to apply ozone to sanitize cheese aging rooms.

The device, consisting of an oxygen concentrator, ozone generator, display with a programmable logic controller (PLC) for remote management, process and safety sensors, Teflon tubing, and wiring (Biofresh Europa S.r.l., Brescia, Italy), was designed to be portable and programmable, complying with current regulations and using CE‐certified materials. Using the oxygen concentrator ensures compliance with the European regulation on biocides and ensures that only ozone is produced as a biocide, avoiding the creation of other by‐products that would be formed by feeding the generator with air. Software was developed to manage ozone concentrations, treatment times, and safety procedures. Functionality tests were carried out to enable remote management via Ethernet connection and a dedicated IP address.

Based on the laboratory test results,^5^, ^18^ the industrial pilot‐scale trial used gaseous ozone at low concentrations during the aging phase (400 ppb, or 0.856 mg m^−3^) for 6 h per day on alternate nights (three times a week, from 10 p.m. to 4 a.m. to ensure operator safety as they are absent from work) (O_3_ sample) compared to untreated cheese sample (control (CTL) sample). The trial lasted 60 days, and ozone treatments were applied until the 40th day of aging (average ripening cycle).

Thirty‐six unmarked cheeses (destined for disposal) produced in the previous 48 h were divided into two aging chambers (60 m^3^ each): traditional (normal air atmosphere, CTL) and innovative (gaseous ozone in air atmosphere, O_3_). Two cheese wheels were processed as the initial time point (T0).

The aging chambers were maintained at 8 °C and 85% relative humidity, following the PDO's standard parameters. Several factors, including material compatibility with gaseous ozone, treatment duration, and impact on daily operations, were considered, and treatments were scheduled when no staff was present and safety measures were in place. The ozone generator was placed outside the treatment area, and the ozone was injected through a Teflon tube. A specific sensor was installed to monitor ozone concentration (model: Ozone Sensor OEM‐3; Biofresh Group Ltd, Stocksfield, UK). Sampling occurred every 10 days (T0, T10, T20, T30, T40, T50, and T60) for weight loss percentage, microbiological analysis, technological analysis, and sensory analysis starting at T30. Chemical analysis was also performed to assess the centesimal composition and peroxide value.

#### Weight loss percentage

Five cheese samples from each treatment were weighed at each sampling time. Weight was measured using a technical balance (Adam Equipment Co., Ltd, Milton Keynes, UK). The percentage of weight loss (WL) was calculated using the following equation:

(1) $$\mathrm{WL}=\left(W₀–W_{t}\right)/W₀\times 100$$

where *W*₀ represents the initial sample mass and *W*_t_ is the sample mass at the considered sampling time.

#### Microbiological analysis

Microbiological analysis was carried out according to UNI EN ISO 4833‐1^19^ for microorganism counts at 30 °C, and CCFRA G43 met. 2.1.1:2007^20^ for yeast and mold counts at each sampling time considered. The analyses were performed in triplicate, and the results were expressed as colony forming units per gram of product on a logarithmic scale (log_10_ CFU g^−1^). Subsamples were taken from the rind area of each cheese.

#### Sensory analysis

After 30 days from the start of the trial (the minimum aging period adopted by the producer), the quality and descriptor of the typicity of this cheese were organoleptically evaluated by a panel of ten internal judges, knowledgeable of the product and trained in the sensory descriptive evaluation, following ISO 13299:2016^21^ and ISO 8586:2023^22^ guidelines. The panelists were instructed on reference parameters, terminology, and sensory evaluation techniques. A tasting sheet was created based on the characteristics described in the official product specifications^11^ and in compliance with ISO 13299:2016^21^ and ISO 4121:2003^23^ standards. The evaluated attributes/descriptors included rind appearance, rind color, paste color, cut appearance, aroma, texture, taste, milk, fresh butter, yogurt, rancid, and spicy. The samples were rated on a scale from 1 (totally unacceptable/absence of the descriptor) to 10 (totally acceptable/intense presence of the descriptor). The test was conducted in a heated/air‐conditioned meeting room with white light, according to ISO 8589:2007.^24^

Initially, the cheeses were evaluated in whole and cut form based on the attributes in the evaluation sheet. The cheese samples were then cut into approximately 15 g cubes and placed on white plastic plates for tasting. The samples were coded for a blind evaluation and served randomly at room temperature (20 °C ± 2 °C). After each sample, the panelists rinsed their mouths with still water and waited 5 min before tasting the following sample.

#### Technological analysis

The cheeses were quickly transported to the Department of Innovation for Biological, Agro‐food and Forestry Systems (DIBAF – University of Tuscia, Viterbo, Italy) and maintained at an appropriate temperature to ensure quality (8 °C ± 2 °C). The cheeses were photographed on a black background using a Canon camera (model EOS 2000D; Canon Inc., Tokyo, Japan) to document and analyze the visual distribution and extent of microbial growth on the surface. The wheels were then cut in half, with one half photographed. The rind thickness was measured for each sample (in millimeters).

The semi‐samples were used for color evaluation with a Minolta colorimeter (Minolta C2500; Konica Minolta, Ramsey, NY, USA). Sixteen random points on both the rind and paste were measured for chromaticity values L* (lightness), a* (green to red), and b* (blue to yellow) for each sample considered. The color difference (ΔE*) was calculated for each sample at each sampling time from the start of the aging phase. The difference can be classified as slight (< 1.5), distinct (1.5–3), or very distinct (> 3),^25^ or imperceptible (ΔE* < 1), minimal (1 ≤ ΔE* < 2), just perceptible (2 ≤ ΔE* < 3), perceptible (3 ≤ ΔE* < 5), strong difference (5 ≤ ΔE* < 12), and a different color (ΔE* ≥12).^26^ Additionally, the white index (WI) and yellow index (YI) were calculated.^25^ The equations for these indices are as follows:

(2) $${\Delta E}^{*}=\sqrt{{\left({\Delta L}^{*}\right)}^{2}+{\left({\Delta a}^{*}\right)}^{2}+{\left({\Delta b}^{*}\right)}^{2}}$$

(3) $$\mathrm{WI}=100-\sqrt{{\left(100-L^{*}\right)}^{2}+a^{*2}+b^{*2}}$$

(4) $$\mathrm{YI}=\frac{142.86b^{*}}{L^{*}}$$

The product's chewability (texture profile analysis (TPA)) was evaluated using compression testing with the Instron Universal Testing Machine (model 4301; Instron Inc., Canton, MS, USA) and expressed in newtons. Eight measurements (for each sample) were taken from the cube‐shaped samples (20 mm × 20 mm × 20 mm), with samples taken from the edge (excluding the rind). The compression test provided rheological parameters describing the cheese structure. Each cube was compressed and released twice by the probe (double compression cycle), simulating chewing action (like jaw movement). The compression cycle was done under specific conditions (compression speed 1 mm s^−1^, 50% compression of the initial height), with a 3‐s wait between compressions. The resistance to compression was recorded in a force/distance curve, from which various parameters were derived to calculate the chewability of the product. These parameters include hardness (in newtons), elasticity (–), cohesiveness (–), adhesiveness (in N mm), and resilience (–). Chewability (in newtons) was obtained by multiplying hardness × cohesiveness × elasticity, representing the force required to chew a solid food until it is ready for swallowing.^27^

#### Chemical analysis

The following chemical analyses were performed: moisture; dry matter (DM); ash content; nitrogen and crude protein content; fat content. Additionally, the peroxide value was assessed.

Moisture (%), DM (%), and ash (%) were determined according to official methods with some modifications.^28^ In brief, 3 g of each sample were weighed and placed in porcelain cups in a 105 °C oven for 12 h to ensure complete moisture removal. After 12 h, the samples were transferred to a desiccator, cooled, and weighed to determine WL. After calculating the WL, the samples were transferred to a muffle furnace at 550 °C for 12 h. They were then cooled in a desiccator and weighed to determine the ash content.

Total nitrogen (TN) and crude protein content (% fresh weight (FW)) were determined by the Kjeldahl method, according to ISO 8968‐1:2014.^29^ A sample of 0.5 g was weighed and introduced into glass test tubes, to which a catalytic tablet and 6 mL of concentrated sulfuric acid (H_2_SO_4_, 95–98%) were added (digestion phase). After cooling, the samples were distilled with 50 mL of sodium hydroxide (NaOH, 30%). After distillation with boric acid (4%) and nitrogen indicator drops, the samples were titrated with hydrochloric acid (HCl, 0.1N).

Fat content was determined using the ISO 3433:2008 – Van Gulik method.^30^ Briefly, 3 g of each sample were finely ground and mixed with 10 mL of H_2_SO_4_ and 1 mL of amyl alcohol in butyrometers. The fat was calculated as a percentage of FW.

For peroxide value, alkaline extraction of the cheese fat was performed with a potassium hydroxide (KOH) solution, following the official AOAC 920.125:2005 method.^31^ The extracted fat was then evaluated for peroxide value according to the AOAC 965.33:2005 method.^32^ Titration of iodine release was performed using 0.01 mol L^−1^ sodium thiosulfate. Triplicate determinations were carried out for all samples. The peroxide value was calculated using the following equation:

(5) $$\begin{array}{l} \text{Peroxide value}\left(\mathrm{meq}.O_{2}\;{\mathrm{kg}}^{-1}\;\mathrm{fat}\right)\\ \;=\left(S\times M\times 1000\right)\mathrm{per}\;\text{gram of test portion} \end{array}$$

where *S* is milliliters of thiosulfate (blank corrected) and *M* is the molarity of thiosulfate solution.

#### Statistical analysis

The data were expressed as the mean ± standard error (SE). Statistical analysis was performed using analysis of variance (ANOVA) and Tukey's test at a 5% significance level (*P* < 0.05), using the DSAASTAT tool in Excel®. This allowed for identifying significant differences between the aging times and different cheese sample types. Significant differences were indicated by distinct letters.

## RESULTS AND DISCUSSION

### Weight loss (%)

Cheeses undergo several biochemical processes during aging (glycolysis, proteolysis, and lipolysis), which contribute to texture and flavor development.^33^ During this phase, WL occurs due to moisture evaporation, which is a normal dehydration process that influences the final texture.^5^ Both samples gradually lose weight over time, with no significant difference (data not shown). Results are in line with laboratory‐scale tests on Pecorino Foggiano and Toma Piemontese PDO cheeses, where no significant difference in WL was observed at the end of the trial.^4^, ^5^

### Microbiological analysis

The evolution of the microflora is shown in Table 1.

**Table 1:** Microbiological analysis (microorganism count at 30 °C, yeast count, and mold count) of Toma Piemontese PDO cheese samples at 0 (T0), 10 (T10), 20 (T20), 30 (T30), 40 (T40), 50 (T50), and 60 (T60) days of aging at 8 °C and 85% relative humidity, in normal atmosphere (CTL) and with gaseous ozone at 400 ppb (O_3_)

Aging time	Sample	Microorganism count at 30 °C	Yeast count	Mold count
T0	CTL	7.76 ± 0.01 (cA)	2.04 ± 0.01 (eA)	1.30 ± 0.01 (gA)
	O_3_	7.78 ± 0.01 (aA)	2.00 ± 0.01 (fA)	1.30 ± 0.01 (gA)
T10	CTL	6.95 ± 0.01 (fA)	6.00 ± 0.02 (dA)	3.70 ± 0.04 (fA)
	O_3_	6.60 ± 0.01 (eB)	4.70 ± 0.03 (dB)	3.00 ± 0.02 (eB)
T20	CTL	7.50 ± 0.02 (eA)	6.20 ± 0.04 (cdA)	4.40 ± 0.01 (eA)
	O_3_	7.30 ± 0.03 (cB)	5.20 ± 0.02 (cB)	3.50 ± 0.02 (bB)
T30	CTL	8.00 ± 0.01 (bA)	6.30 ± 0.01 (cA)	6.30 ± 0.02 (dA)
	O_3_	7.48 ± 0.02 (bB)	6.00 ± 0.01 (bB)	4.11 ± 0.03 (aB)
T40	CTL	8.00 ± 0.04 (bA)	7.60 ± 0.02 (aA)	7.30 ± 0.04 (aA)
	O_3_	7.48 ± 0.02 (bB)	3.57 ± 0.05 (eB)	2.00 ± 0.02 (fB)
T50	CTL	7.60 ± 0.02 (dA)	7.00 ± 0.01 (bA)	6.48 ± 0.03 (cA)
	O_3_	7.00 ± 0.03 (dB)	6.95 ± 0.01 (aB)	3.08 ± 0.01 (dB)
T60	CTL	8.30 ± 0.02 (aA)	7.48 ± 0.01 (aA)	7.08 ± 0.02 (bA)
	O_3_	7.34 ± 0.03 (cB)	6.90 ± 0.01 (aB)	3.30 ± 0.04 (cB)

*Note*: The data are the mean ± standard error of three replicates and are expressed in log_10_ CFU g^−1^. Different lowercase letters in the same column for each sample type indicate significant differences over the aging period (*P* < 0.05). Different uppercase letters in the same column at the same aging time indicate significant differences between cheeses with different treatments (*P* < 0.05).

The results show a significant increase in the general microflora over time for the CTL sample, while a decrease in mesophilic microorganisms is observed for the O_3_ sample. The effectiveness of the gaseous ozone treatment is evident from the first 10 days of aging (T10), with significant reductions in mesophilic counts, yeasts, and molds at each sampling time compared to the CTL sample. At the medium aging time (T40), the O_3_ sample shows a significant reduction of about −4 log_10_ CFU g^−1^ for yeasts and −5 log_10_ CFU g^−1^ for molds concerning the CTL sample. After stopping the ozone treatments, the fermentative activity in the O_3_ sample reduces the difference in yeast counts compared to the CTL sample, but a significant increase in mold counts is also observed (T50 and T60), indicating an inhibitory, non‐destructive effect of ozone, as previously observed by Gibson *et al*.^34^ and confirmed more recently by Tabla and Roa.^3^ This confirms the different sensitivity of molds to gaseous ozone, which are more resistant than bacteria.^35^ It is important to note that, after the treatment interruption period (T40), the microbial load in the O_3_ sample (T60) reaches values comparable to the CTL sample from day 10 of aging (T10), potentially extending the product's shelf life. Our results demonstrate the efficacy of gaseous ozone in controlling microflora over time and are consistent with previous laboratory‐scale test^5^ and Pecorino Foggiano cheese,^4^ Ricotta Salata di Pecora, Gorgonzola, Taleggio,^36^ and soft cheese Torta del Casar^3^ studies.

### Sensory analysis

Table 2 summarizes the organoleptic evaluation of Toma Piemontese starting from 30 days of aging. The acceptability of the cheese in terms of paste color, cut appearance, and texture was not affected by the gaseous ozone treatment, showing no significant differences compared to the CTL sample at any aging time. However, significantly higher acceptability values were observed for the O_3_ sample regarding rind appearance and color from the first tasting time. Sensory scores for Torta del Casar cheese^3^ also showed higher ratings for rind appearance and color, indicating that gaseous ozone treatment resulted in a cleaner surface. The O_3_ sample was significantly more acceptable regarding aroma from 40 days of aging and taste at 30 and 50 days. Consumer tests conducted for the laboratory‐scale test previously demonstrated greater acceptability for the O_3_ sample at 60 days of aging.^5^

**Table 2:** Sensory analysis – evaluation of attributes (1 – totally unacceptable; 10 – totally acceptable) for Toma Piemontese PDO cheese samples at 30 (T30), 40 (T40), 50 (T50), and 60 (T60) days of aging at 8 °C and 85% relative humidity in typical atmosphere (CTL) and with gaseous ozone at 400 ppb (O_3_)

Aging time	Sample	Rind appearance	Rind color	Paste color	Cut appearance	Aroma	Texture	Taste
T30	CTL	4.70 ± 0.30 (aB)	4.40 ± 0.27 (aB)	7.20 ± 0.20 (aA)	6.80 ± 0.20 (aA)	6.90 ± 0.28 (aA)	7.40 ± 0.16 (aA)	6.70 ± 0.26 (abB)
	O_3_	7.30 ± 0.21 (aA)	7.30 ± 0.21 (aA)	7.10 ± 0.10 (aA)	7.20 ± 0.20 (aA)	7.10 ± 0.10 (aA)	7.10 ± 0.18 (aA)	7.50 ± 0.22 (aA)
T40	CTL	5.10 ± 0.53 (aB)	4.90 ± 0.46 (aB)	7.60 ± 0.16 (aA)	6.70 ± 0.30 (aA)	6.70 ± 0.33 (aB)	7.20 ± 0.13 (aA)	7.40 ± 0.27 (aA)
	O_3_	7.00 ± 0.21 (aA)	7.40 ± 0.16 (aA)	7.50 ± 0.17 (aA)	7.30 ± 0.15 (aA)	7.60 ± 0.16 (aA)	7.20 ± 0.13 (aA)	7.40 ± 0.31 (aA)
T50	CTL	5.90 ± 0.43 (aB)	5.90 ± 0.0.46 (aB)	7.30 ± 0.21 (aA)	7.30 ± 0.15 (aA)	6.60 ± 0.30 (aB)	7.30 ± 0.15 (aA)	6.30 ± 0.30 (bB)
	O_3_	7.50 ± 0.17 (aA)	7.70 ± 0.15 (aA)	7.50 ± 0.22 (aA)	7.20 ± 0.20 (aA)	7.40 ± 0.16 (aA)	7.20 ± 0.13 (aA)	7.50 ± 0.22 (aA)
T60	CTL	6.10 ± 0.31 (aB)	5.90 ± 0.38 (aB)	7.30 ± 0.15 (aA)	6.90 ± 0.18 (aA)	6.80 ± 0.13 (aB)	7.00 ± 0.15 (aA)	7.30 ± 0.15 (aA)
	O_3_	7.60 ± 0.16 (aA)	7.80 ± 0.13 (aA)	7.20 ± 0.13 (aA)	7.40 ± 0.16 (aA)	7.60 ± 0.13 (aA)	7.20 ± 0.13 (aA)	7.70 ± 0.15 (aA)

*Note*: The data are the mean ± standard error of the ten judges. Different lowercase letters in the same column for each sample type indicate significant differences over the aging time (*P* < 0.05). Different uppercase letters in the same column at the same aging time indicate significant differences between cheeses with different treatments (*P* < 0.05).

Table 3 shows the perception of different descriptors. The most prominent descriptors for both samples at each tasting time were fresh milk and butter, with higher values for the O_3_ sample at T40 compared to the CTL sample. The presence of milky notes aligns with the organoleptic characteristics outlined in the production specifications and the laboratory‐scale test results.^5^, ^11^, ^18^ No significant differences were observed for yogurt notes between the samples at various times. Both rancid and spicy notes were almost absent in both samples, as demonstrated in the laboratory tests and gas chromatography analysis.^5^, ^18^ This suggests that ozone treatment does not negatively impact the quality of the cheese. Morandi *et al*.,^36^ in their study on the aroma compounds in the rind area of Taleggio PDO cheese, highlighted that ozone treatment inhibits lipolysis, which was also observed in Gorgonzola PDO cheese. Lipolysis can contribute to lipid peroxidation, which leads to the formation of various compounds such as aldehydes, ketones, and acids, which are typically responsible for undesirable odors and flavors in food.^37^ Furthermore, compounds like aldehydes (e.g., hexanal and nonanal) and ketones (e.g., 2‐heptanone) contribute to pungent and rancid flavors.^38^, ^39^

**Table 3:** Sensory analysis – evaluation of descriptors (1 – total absence; 10 – intense presence) for Toma Piemontese PDO cheese samples at 30 (T30), 40 (T40), 50 (T50), and 60 (T60) days of aging at 8 °C and 85% relative humidity, under normal atmosphere (CTL) and with gaseous ozone at 400 ppb (O_3_)

Aging time	Sample	Milk	Fresh butter	Yogurt	Rancid	Spicy
T30	CTL	7.00 ± 0.21 (aA)	7.00 ± 0.21 (aA)	4.50 ± 0.37 (aA)	1.40 ± 0.16 (aA)	1.20 ± 0.13 (abA)
	O_3_	7.10 ± 0.23 (aA)	7.40 ± 0.22 (aA)	4.70 ± 0.42 (aA)	1.50 ± 0.17 (aA)	1.30 ± 0.15 (aA)
T40	CTL	6.20 ± 0.25 (abB)	6.00 ± 0.33 (abB)	3.20 ± 0.59 (aA)	1.50 ± 0.27 (aA)	1.60 ± 0.27 (aA)
	O_3_	7.10 ± 0.23 (aA)	7.30 ± 0.30 (aA)	3.10 ± 0.55 (bA)	1.50 ± 0.27 (aA)	1.50 ± 0.27 (aA)
T50	CTL	5.40 ± 0.16 (bA)	5.10 ± 0.31 (bA)	3.90 ± 0.23 (aA)	1.10 ± 0.10 (aA)	1.00 ± 0.00 (bA)
	O_3_	5.50 ± 0.22 (bA)	5.30 ± 0.21 (bA)	4.00 ± 0.33 (abA)	1.10 ± 0.10 (aA)	1.00 ± 0.00 (aA)
T60	CTL	6.10 ± 0.28 (bA)	5.90 ± 0.23 (bA)	3.20 ± 0.36 (aA)	1.00 ± 0.00 (aA)	1.00 ± 0.00 (bA)
	O_3_	6.50 ± 0.31 (aA)	6.50 ± 0.34 (aA)	3.00 ± 0.33 (bA)	1.00 ± 0.00 (aA)	1.00 ± 0.00 (aA)

*Note*: The data are the mean ± standard error of the ten judges. Different lowercase letters in the same column for each sample type indicate significant differences over the aging period (*P* < 0.05). Different uppercase letters in the same column at the same aging time indicate significant differences between cheeses with different treatments (*P* < 0.05).

### Technological analysis

Visual appearance and texture are key quality indicators for consumers when making purchasing decisions. Ensuring a consistently high standard across the entire production chain is a significant challenge for PDO cheeses. Color can serve as a key quality indicator, reflecting attributes like flavor and maturation, and can be objectively evaluated using the tristimulus colorimetric method.^5^

**Figure 1:**
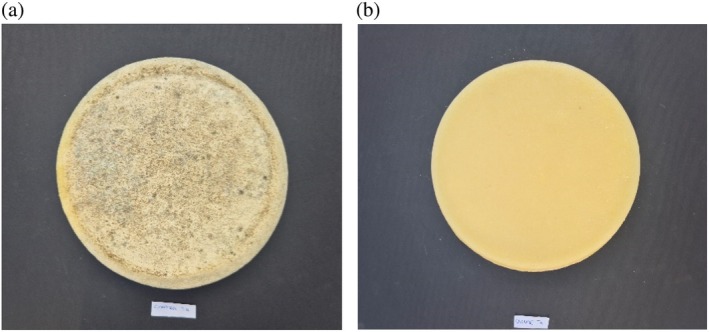
Development of surface microflora for CTL (a) and O_3_ (b) samples at the commercial optimum – 40 days of aging (T40) – Toma Piemontese PDO cheese.

Figure 1(a),(b) shows the visual progression of microflora at T40, the optimal commercial aging time, for both the CTL and O_3_ samples.

**Figure 2:**
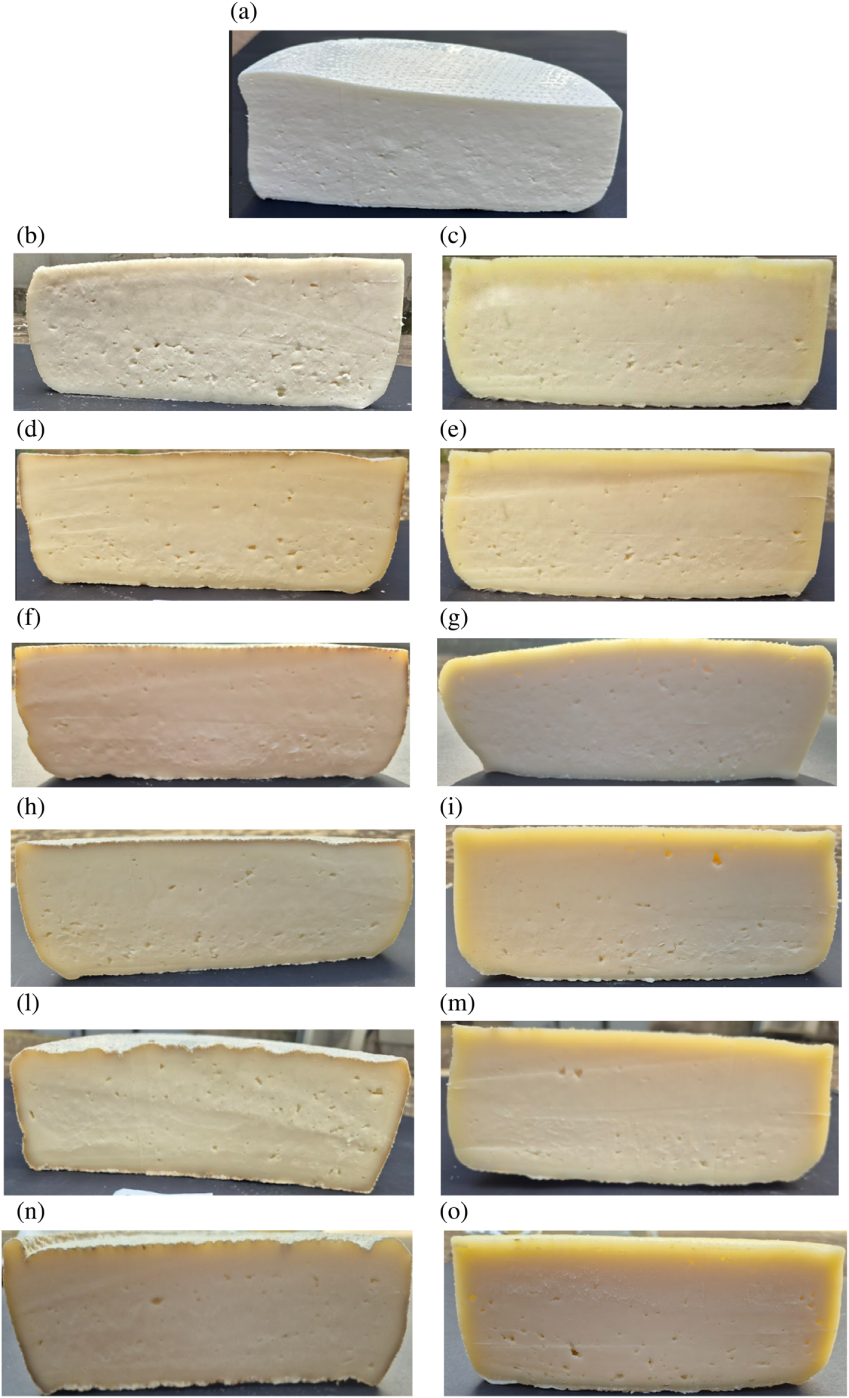
Average rind thickness for the CTL sample at (a) 0 (T0), (b) 10 (T10), (d) 20 (T20), (f) 30 (T30), (h) 40 (T40), (l) 50 (T50), and (n) 60 (T60) days of aging and for the O_3_ sample at (a) 0 (T0), (c) 10 (T10), (e) 20 (T20), (g) 30 (T30), (i) 40 (T40), (m) 50 (T50), and (o) 60 (T60) days of aging – Toma Piemontese PDO cheese.

The average rind thickness for the CTL and O_3_ samples can be observed in Fig. 2(a)–(o). The average values for the CTL sample at T0, T10, T20, T30, T40, T50, and T60 were as follows: 0 mm, 0 mm, 2 mm, 2 mm, 2 mm, 3 mm, and 3 mm. For the O_3_ sample, the values were: 0 mm, 1 mm, 2 mm, 3 mm, 4 mm, 4 mm, and 4 mm. The rind of the O_3_ sample is only slightly thicker, which may be attributed to the reduction in microflora on the surface. This reduction could have subtly influenced the natural moisture balance between the surface and the core of the cheese, leading to minor differences in rind formation.

Table 4 shows the colorimetric data of the rind. The L* value tends to decrease over time for both samples, with lower values for the O_3_ sample. A decrease in external brightness was also observed in studies by Sánchez‐Macías *et al*.^40^ on goat cheese and by Pinho *et al*.^41^ on Terrincho sheep cheese. However, L* values tend to equalize at the minimum aging time for the commercial sale of Toma Piemontese (T30), showing no significant difference. The a* values significantly differ between samples at every aging time, with CTL sample tending toward red (+*a*). Red‐smear soft cheeses, such as Tilsit, are typically characterized by red to brown‐orange surfaces, a coloration resulting from the development of specific microflora. This category also includes Taleggio, which is characterized by positive a* and b* values.^42^ Our results show that the b* values trend toward yellow (+*b*) for both samples, with no significant differences at 30, 40, and 50 days of aging. Lower yellow values were observed at 60 days of aging for the O_3_ sample. The ΔE* values exceed 12 for both samples at all aging times, indicating color differences from the start of the trial. WI tends to decrease over time for both samples, while YI increases, with no significant difference at the minimum aging time for sale (T30).

**Table 4:** Color of the rind (L*, a*, b*, ΔE*, WI, YI) for Toma Piemontese PDO cheese samples at 0 (T0), 10 (T10), 20 (T20), 30 (T30), 40 (T40), 50 (T50), and 60 (T60) days of aging at 8 °C and 85% relative humidity in normal atmosphere (CTL) and with gaseous ozone at 400 ppb (O_3_)

Aging time	Sample	L*	a*	b*	ΔE*	WI	YI
T0	CTL	85.41 ± 0.50 (aA)	−1.06 ± 0.06 (cA)	9.50 ± 0.11 (cA)	–	82.53 ± 0.44 (aA)	15.90 ± 0.24 (cA)
	O_3_	85.41 ± 0.50 (aA)	−1.06 ± 0.06 (bcA)	9.50 ± 0.11 (dA)	–	82.53 ± 0.44 (aA)	15.90 ± 0.24 (cA)
T10	CTL	73.05 ± 1.17 (bA)	0.26 ± 0.13 (bA)	13.54 ± 0.28 (aB)	13.21 ± 1.10 (bA)	69.74 ± 1.03 (bA)	26.55 ± 0.60 (aB)
	O_3_	71.71 ± 1.73 (bA)	−1.03 ± 0.06 (abcB)	14.57 ± 0.19 (aA)	14.83 ± 1.61 (dA)	68.01 ± 1.55 (bA)	29.30 ± 0.85 (aA)
T20	CTL	71.15 ± 1.76 (bcA)	0.11 ± 0.25 (bA)	9.50 ± 0.37 (cB)	14.42 ± 1.75 (abB)	68.58 ± 1.76 (bA)	19.49 ± 1.24 (bcB)
	O_3_	63.26 ± 1.69 (cdB)	−0.66 ± 0.16 (abB)	12.12 ± 0.46 (bA)	22.38 ± 1.69 (bcA)	61.24 ± 1.65 (cdB)	27.80 ± 1.50 (aA)
T30	CTL	65.67 ± 1.50 (cA)	1.29 ± 0.21 (aA)	11.99 ± 0.27 (bA)	20.05 ± 1.52 (aA)	63.58 ± 1.47 (cA)	26.41 ± 1.05 (aA)
	O_3_	67.11 ± 1.53 (bcA)	−0.55 ± 0.08 (aB)	11.27 ± 0.38 (bcA)	18.44 ± 1.54 (cdA)	65.19 ± 1.52 (bcA)	24.34 ± 1.21 (abA)
T40	CTL	68.19 ± 0.91 (bcA)	0.65 ± 0.29 (abA)	10.33 ± 0.46 (cA)	17.46 ± 0.91 (abB)	66.46 ± 0.87 (bcA)	21.70 ± 1.00 (bB)
	O_3_	53.68 ± 1.89 (eB)	−0.77 ± 0.12 (abcB)	10.28 ± 0.32 (cdA)	31.77 ± 1.89 (aA)	52.51 ± 1.87 (eB)	28.14 ± 1.74 (aA)
T50	CTL	67.69 ± 1.76 (bcA)	−0.14 ± 0.23 (bA)	10.61 ± 0.24 (cA)	17.86 ± 1.74 (abB)	65.89 ± 1.65 (bcA)	22.61 ± 0.73 (bB)
	O_3_	54.54 ± 1.03 (eB)	−0.92 ± 0.18 (abcB)	10.15 ± 0.37 (cdA)	30.91 ± 1.04 (aA)	53.39 ± 1.05 (eB)	26.87 ± 1.34 (aA)
T60	CTL	68.06 ± 1.06 (bcA)	0.30 ± 0.17 (bA)	10.73 ± 0.26 (bcA)	17.49 ± 1.05 (abB)	66.26 ± 1.01 (bcA)	22.62 ± 1.05 (bA)
	O_3_	57.38 ± 1.13 (deB)	−1.26 ± 0.11 (cB)	7.83 ± 0.26 (eB)	28.11 ± 1.12 (abA)	56.63 ± 1.13 (deB)	19.68 ± 0.82 (bcB)

*Note*: The data are the mean ± standard error of 16 replicates. Different lowercase letters within the same column for each sample type indicate significant differences over the aging time (*P* < 0.05). Different uppercase letters within the same column at the same aging time indicate significant differences between cheeses with different treatments (*P* < 0.05). ΔE*, color difference; WI, white index; YI, yellow index.

Regarding data on paste color (Table 5), L* values tend to decrease over time for both samples, with no significant differences at T40. This decrease in L* may be due to increased protein hydration and reduced light diffusion associated with free water.^40^ Similar results were observed in Asiago^43^ and Montasio^44^ cheeses. The negative a* values (green trend) tend to significantly decrease at the commercial minimum and optimum of aging (T30 and T40), while b* values (yellow trend) increase for both samples over time, showing no significant difference at the commercial optimum (T40), also for ΔE* values, WI and YI.

**Table 5:** Paste color (L*, a*, b*, ΔE*, WI, YI) of Toma Piemontese PDO cheese samples at 0 (T0), 10 (T10), 20 (T20), 30 (T30), 40 (T40), 50 (T50), and 60 (T60) days of aging at 8 °C and 85% relative humidity in normal atmosphere (CTL) and with gaseous ozone at 400 ppb (O3)

Aging time	Sample	L*	a*	b*	ΔE*	WI	YI
T0	CTL	85.48 ± 0.44 (aA)	−1.22 ± 0.04 (bcA)	9.00 ± 0.21 (cA)	—	82.84 ± 0.40 (aA)	15.06 ± 0.37 (cA)
	O_3_	85.48 ± 0.44 (aA)	−1.22 ± 0.04 (bA)	9.00 ± 0.21 (dA)	—	82.84 ± 0.40 (aA)	15.06 ± 0.37 (eA)
T10	CTL	79.24 ± 1.29 (bA)	−1.19 ± 0.07 (bcA)	10.80 ± 0.22 (aA)	6.63 ± 1.26 (aA)	76.50 ± 1.22 (bA)	19.63 ± 0.73 (aA)
	O_3_	79.81 ± 0.42 (cdA)	−1.21 ± 0.46 (bA)	10.37 ± 0.17 (abcA)	5.87 ± 0.43 (abA)	77.26 ± 0.42 (cdA)	5.87 ± 0.43 (abcA)
T20	CTL	80.98 ± 0.50 (bA)	−1.36 ± 0.06 (cA)	10.70 ± 0.15 (abA)	4.90 ± 0.47 (aB)	78.11 ± 0.46 (bA)	18.89 ± 0.32 (abB)
	O_3_	77.68 ± 1.03 (dB)	−1.32 ± 0.06 (bA)	11.00 ± 0.18 (aA)	8.08 ± 1.03 (aA)	75.05 ± 0.99 (dB)	20.34 ± 0.60 (aA)
T30	CTL	81.67 ± 0.54 (bB)	−0.79 ± 0.04 (aA)	10.24 ± 0.15 (abA)	4.15 ± 0.51 (aA)	78.96 ± 0.51 (bB)	17.94 ± 0.55 (abA)
	O_3_	83.06 ± 0.26 (abA)	−0.72 ± 0.04 (aA)	9.69 ± 0.10 (cdB)	2.63 ± 0.24 (cB)	80.46 ± 0.24 (abA)	16.66 ± 0.19 (deB)
T40	CTL	80.43 ± 0.58 (bA)	−0.69 ± 0.04 (aA)	10.02 ± 0.15 (bA)	5.21 ± 0.59 (aA)	77.98 ± 0.56 (bA)	17.84 ± 0.37 (bA)
	O_3_	81.31 ± 0.42 (bcA)	−0.76 ± 0.03 (aA)	9.85 ± 0.13 (bcA)	4.31 ± 0.42 (bcA)	78.85 ± 0.40 (bcA)	17.33 ± 0.30 (cdA)
T50	CTL	79.91 ± 0.42 (bA)	−1.08 ± 0.04 (bA)	10.74 ± 0.10 (aA)	5.86 ± 0.41 (aA)	77.18 ± 0.38 (bA)	19.22 ± 0.23 (abA)
	O_3_	79.09 ± 1.15 (cdA)	−0.73 ± 0.07 (aB)	9.91 ± 0.23 (bcB)	6.56 ± 1.14 (abA)	76.79 ± 1.10 (cdA)	18.00 ± 0.62 (bcdA)
T60	CTL	81.36 ± 0.56 (bA)	−1.27 ± 0.07 (bcA)	10.45 ± 0.10 (abA)	4.45 ± 0.53 (aB)	78.58 ± 0.50 (bA)	18.36 ± 0.23 (abB)
	O_3_	76.99 ± 0.56 (dB)	−1.29 ± 0.05 (bA)	10.44 ± 0.17 (abA)	8.63 ± 0.57 (aA)	74.69 ± 0.56 (dB)	19.41 ± 0.45 (abA)

*Note*: The data are the mean ± standard error of 16 replicates. Different lowercase letters in the same column for each sample type indicate significant differences over the aging period (*P* < 0.05). Different uppercase letters in the same column at the same aging time indicate significant differences between cheeses with different treatments (*P* < 0.05). ΔE*, color difference; WI, white index; YI, yellow index.

Table 6 shows the TPA results. The hardness generally increases over time for both samples, more so for the O_3_ sample. The milk coagulation process, curd cutting, whey drainage, and compression influence the compactness and homogeneity of the cheese, affecting its rheological properties^41^ and thus its texture, including hardness. Elasticity, however, tends to decrease significantly for both samples over time, with lower elasticity observed at 30 and 40 days for the O_3_ sample compared to the CTL sample. Cohesiveness also decreases significantly over time for both samples, while adhesiveness values are similar for the CTL sample over time, unlike the O_3_ sample, where adhesiveness trends towards increasingly negative values, although no significant differences are observed with respect to CTL sample at the commercial optimum (T30 and T40). Resilience significantly decreases over time for both samples, with significantly higher negative values for the CTL sample at 30 and 40 days compared to O_3_ sample. Lower resilience values during 60 days of aging were also observed in Terrincho sheep cheese.^41^ Finally, chewiness value tends to decrease significantly for both samples up to 40 days of aging, with significantly lower values for the O_3_ sample at T30 (more chewable) and no significant differences at T40. At T60, the O_3_ sample shows a higher value than the CTL sample, but not significantly different from the start of the trial. A similar trend was observed in the laboratory‐scale test.^5^ Overall, similar texture changes during aging have been observed by Segura *et al*.^45^

**Table 6:** Texture profile analysis (TPA – hardness, elasticity, cohesiveness, adhesiveness, resilience, and chewiness) of Toma Piemontese PDO cheese samples at 0 (T0), 10 (T10), 20 (T20), 30 (T30), 40 (T40), 50 (T50), and 60 (T60) days of aging at 8 °C and 85% relative humidity in normal atmosphere (CTL) and with gaseous ozone at 400 ppb (O_3_)

Aging time	Sample	Hardness (N)	Elasticity (–)	Cohesiveness (–)	Adhesiveness (N mm)	Resilience (–)	Chewinees (N)
T0	CTL O_3_	14.85 ± 1.85 (bcA) 14.85 ± 1.85 (cA)	1.03 ± 0.07 (aA) 1.03 ± 0.07 (aA)	0.78 ± 0.01 (aA) 0.78 ± 0.01 (aA)	−15.98 ± 2.33 (abA) −15.98 ± 2.33 (abA)	−0.38 ± 0.01 (dA) −0.38 ± 0.01 (eA)	11.33 ± 0.68 (aA) 11.33 ± 0.68 (aA)
T10	CTL	19.04 ± 1.26 (abA)	0.52 ± 0.04 (cA)	0.59 ± 0.03 (cdA)	−15.37 ± 2.27 (abA)	−0.17 ± 0.02 (aA)	5.85 ± 0.65 (cA)
	O_3_	23.12 ± 2.76 (bA)	0.54 ± 0.04 (bcA)	0.57 ± 0.01 (dA)	−17.21 ± 1.80 (abA)	−0.16 ± 0.01 (bcA)	6.89 ± 0.74 (bA)
T20	CTL	15.44 ± 0.57 (bcB)	0.74 ± 0.03 (bA)	0.71 ± 0.01 (abcA)	−15.48 ± 0.68 (abA)	−0.27 ± 0.00 (bcA)	8.05 ± 0.35 (bcB)
	O_3_	22.92 ± 1.87 (bA)	0.70 ± 0.05 (bA)	0.68 ± 0.02 (bA)	−21.89 ± 1.74 (bcB)	−0.25 ± 0.02 (dA)	10.63 ± 0.66 (aA)
T30	CTL	10.73 ± 0.82 (cB)	0.99 ± 0.03 (aA)	0.73 ± 0.01 (abA)	−10.21 ± 0.94 (aA)	−0.31 ± 0.01 (cB)	7.56 ± 0.40 (bcA)
	O_3_	13.84 ± 0.51 (cA)	0.67 ± 0.04 (bcB)	0.59 ± 0.01 (cdB)	−10.78 ± 0.32 (aA)	−0.17 ± 0.02 (bcA)	5.52 ± 0.36 (bB)
T40	CTL	16.36 ± 1.26 (abcB)	0.67 ± 0.03 (bcA)	0.61 ± 0.02 (bcdA)	−14.25 ± 1.21 (aA)	−0.21 ± 0.02 (abB)	6.58 ± 0.44 (cA)
	O_3_	21.70 ± 0.96 (bcA)	0.51 ± 0.01 (cB)	0.66 ± 0.01 (bcA)	−11.93 ± 1.11 (aA)	−0.10 ± 0.01 (aA)	7.25 ± 0.25 (bA)
T50	CTL	23.05 ± 2.78 (aB)	0.65 ± 0.03 (bcA)	0.68 ± 0.02 (abcA)	−23.14 ± 2.88 (bA)	−0.24 ± 0.02 (bA)	9.98 ± 1.08 (abA)
	O_3_	34.38 ± 1.63 (aA)	0.64 ± 0.02 (bcA)	0.59 ± 0.02 (cdB)	−28.68 ± 2.18 (cA)	−0.20 ± 0.01 (cdA)	13.21 ± 1.08 (aA)
T60	CTL	21.36 ± 1.38 (abB)	0.56 ± 0.03 (bcA)	0.49 ± 0.07 (dB)	−17.01 ± 1.28 (abA)	−0.16 ± 0.02 (aA)	5.85 ± 1.09 (cB)
	O_3_	39.59 ± 2.38 (aA)	0.50 ± 0.02 (cA)	0.65 ± 0.01 (bcA)	−29.75 ± 3.46 (cB)	−0.13 ± 0.01 (abA)	12.68 ± 0.91 (aA)

*Note*: The data are the mean ± standard error of eight replicates. Different lowercase letters in the same column for each sample type indicate significant differences over the aging period (*P* < 0.05). Different uppercase letters in the same column at the same aging time indicate significant differences between cheeses with different treatments (*P* < 0.05).

### Chemical analysis

Table 7 shows the centesimal composition of Toma Piemontese cheese samples during aging. Moisture significantly decreases over time for both samples, with no significant differences observed at T30 and T40. Similarly, Tabla and Roa^3^ found a significant decrease in moisture over time, with no significant difference in moisture content between control and ozone‐treated (2 ppm) Torta del Casar cheese. This decrease is due to the natural dehydration process during cheese maturation.^18^ Ash content also significantly decreases, while fat tends to increase without significant differences between samples. Fat content (% DM) aligns with the minimum fat percentage in the production specification.^11^ Protein content tends to increase over time for the O_3_ sample, as observed in the laboratory‐scale test.^18^ For the CTL sample, no differences were observed at the end of aging compared to T0. The increase in crude protein content is associated with higher proteolytic activity, as noted by Arlorio *et al*.^46^ and Bertolino *et al*.^47^ for Toma cheese. Protein and fat concentrations may be influenced by water loss, as seen in Segato *et al*.^48^ Marcos *et al*.^49^ also found an inverse correlation between proteolysis and ash concentration, with the latter decreasing.

**Table 7:** Centesimal composition (moisture, ash, fat, and protein) of Toma Piemontese PDO cheese samples at 0 (T0), 10 (T10), 20 (T20), 30 (T30), 40 (T40), 50 (T50), and 60 (T60) days of aging at 8 °C and 85% relative humidity in typical atmosphere (CTL) and with gaseous ozone at 400 ppb (O_3_)

Aging time	Sample	Moisture (%)	Ash (%)	Fat (% FW)	Fat (% DM)	Protein (% FW)
T0	CTL O_3_	51.10 ± 0.21 (aA) 51.10 ± 0.21 (aA)	8.43 ± 0.20 (aA) 8.43 ± 0.20 (aA)	20.00 ± 0.00 (cA) 20.00 ± 0.00 (cA)	40.90 ± 0.18 (bcA) 40.90 ± 0.18 (cA)	20.75 ± 0.56 (cdA) 20.75 ± 0.56 (bA)
T10	CTL	51.23 ± 0.10 (aA)	7.47 ± 0.01 (abA)	20.00 ± 0.00 (cA)	41.01 ± 0.08 (bcA)	20.36 ± 0.07 (cdB)
	O_3_	49.46 ± 0.17 (bB)	7.27 ± 0.02 (cdB)	20.00 ± 0.00 (cA)	39.57 ± 0.13 (dB)	23.30 ± 0.08 (abA)
T20	CTL	49.75 ± 0.89 (aA)	7.76 ± 0.13 (aA)	20.00 ± 0.00 (cA)	39.81 ± 0.70 (cA)	22.96 ± 0.26 (abB)
	O_3_	46.40 ± 0.12 (cdA)	7.11 ± 0.03 (cdB)	20.00 ± 0.00 (cA)	37.31 ± 0.08 (eA)	26.39 ± 0.28 (aA)
T30	CTL	47.30 ± 0.02 (abA)	8.49 ± 0.31 (aA)	23.00 ± 0.00 (bA)	43.64 ± 0.02 (abcA)	19.93 ± 0.35 (dA)
	O_3_	47.20 ± 0.00 (cA)	7.81 ± 0.04 (abcA)	23.00 ± 0.00 (bA)	43.56 ± 0.00 (bB)	21.13 ± 0.38 (bA)
T40	CTL	46.27 ± 0.11 (abA)	6.51 ± 0.02 (bB)	25.00 ± 0.00 (aA)	46.53 ± 0.09 (aA)	21.69 ± 0.02 (bcA)
	O_3_	45.45 ± 0.38 (dA)	8.31 ± 0.10 (abA)	25.00 ± 0.00 (aA)	45.83 ± 0.32 (aA)	21.39 ± 0.69 (bA)
T50	CTL	43.80 ± 2.62 (bA)	7.56 ± 0.33 (abA)	25.00 ± 0.00 (aA)	44.48 ± 2.08 (abA)	23.37 ± 0.14 (aA)
	O_3_	42.50 ± 0.05 (eA)	6.95 ± 0.27 (dA)	25.00 ± 0.00 (aA)	43.48 ± 0.04 (bA)	25.60 ± 1.21 (aA)
T60	CTL	45.68 ± 0.15 (abA)	6.65 ± 0.08 (bB)	25.00 ± 0.00 (aA)	46.02 ± 0.13 (aA)	21.16 ± 0.15 (cdB)
	O_3_	42.27 ± 0.29 (eB)	7.54 ± 0.14 (bcdA)	25.00 ± 0.00 (aA)	43.31 ± 0.21 (bB)	25.66 ± 0.12 (aA)

*Note*: The data are the mean ± standard error of three replicates. Different lowercase letters in the same column for each sample type indicate significant differences over the aging time (*P* < 0.05). Different uppercase letters in the same column at the same aging time indicate significant differences between cheeses with different treatments (*P* < 0.05). DM, dry matter; FW, fresh weight.

**Figure 3:**
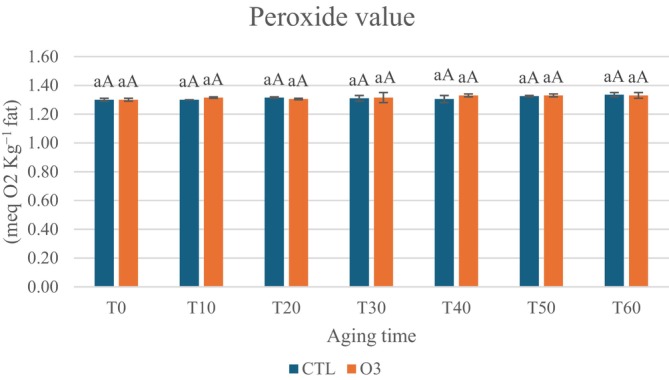
Peroxide value of Toma Piemontese PDO cheese samples at 0 (T0), 10 (T10), 20 (T20), 30 (T30), 40 (T40), 50 (T50), and 60 (T60) days of aging at 8 °C and 85% relative humidity in normal atmosphere (CTL) and with gaseous ozone at 400 ppb (O_3_). Different lowercase letters indicate significant differences over aging time for each sample type (*P* < 0.05). Different uppercase letters at each aging time indicate significant differences between cheeses with different treatments (*P* < 0.05).

Ozone is classified as a reactive oxygen species (ROS). Lipid degradation is primarily caused by oxidation, a complex process involving the reaction between unsaturated fatty acids and molecular oxygen, resulting in the formation of hydroperoxides and peroxides, which are primary compounds. These are unstable and odorless, decomposing quickly into secondary compounds such as aldehydes, ketones, alcohols, esters, and acids that produce undesirable odors and flavors, though their impact on overall aroma perception varies.^39^ An increase in peroxide value indicates the deterioration of dairy product quality during storage.^50^ The results show consistent values throughout aging for both samples, ranging between 1.30 and 1.35 (no significant difference) (Fig. 3). The values found are within the range observed by Kristensen *et al*.^51^ and Mele *et al*.^52^ The peroxide values do not vary significantly, likely because peroxide formation is associated with secondary oxidation products, as noted by Branciari *et al*.^53^

## CONCLUSION

Using low gaseous ozone levels in a cheese industrial pilot scale resulted in a microbiologically and visually improved product, with a significant reduction in microflora starting from the first 10 days of aging. Generally, ozone treatment does not negatively impact the overall quality of Toma Piemontese PDO cheese. Ozone‐treated cheese maintained or improved sensory acceptance, with a cleaner rind and enhanced milky notes. No lipid peroxidation issues were detected, as confirmed by stable peroxide values. These findings support the potential of low‐concentration gaseous ozone in an industrial setting as an effective solution for controlling spoilage microflora, particularly molds, which can impact the product's quality, shelf life, and marketability. In conclusion, this first pilot‐scale application for industrial upgrading highlights how ozone technology can improve hygiene and food safety conditions in the dairy industry, with potential environmental and economic sustainability benefits.

## CONFLICT OF INTEREST

The authors confirm that no conflict of interest exists.
